# Isolation and genomic analysis of 11-aminoundecanoic acid-degrading bacterium *Pseudomonas* sp. JG-B from nylon 11 enrichment culture

**DOI:** 10.7150/jgen.42095

**Published:** 2020-01-25

**Authors:** Jocelyn Gatz-Schrupp, Peter Deckard, Benjamin Hufford, Steven Ly, Peter Tupa, Hisako Masuda

**Keywords:** Nylon 11, biodegradation, genome, Pseudomonas, 11-aminoundecanoic acid, hydrolases.

## Abstract

Nylon 11 is a polymer synthesized from 11-aminoundecanoic acid, and widely used in commercial manufacturing. In this study, we describe the isolation of the first organism capable of metabolizing 11-aminoundecanoic acid from nylon 11 enrichment culture. The strain shows rapid growth on 11-aminoundecanoic acid as a sole source of carbon, nitrogen, and energy. Furthermore, the genome sequence of strain JG-B was deciphered and shown to belong to genus *Pseudomonas*. Many genes encoding putative extracellular hydrolases, as well as homologues of nylon 6 hydrolases (NylB and NylA) were identified, suggesting the metabolic versatility and possibility that this organism could also depolymerase nylon 11 polymers.

## Introduction

**Figure 1:**
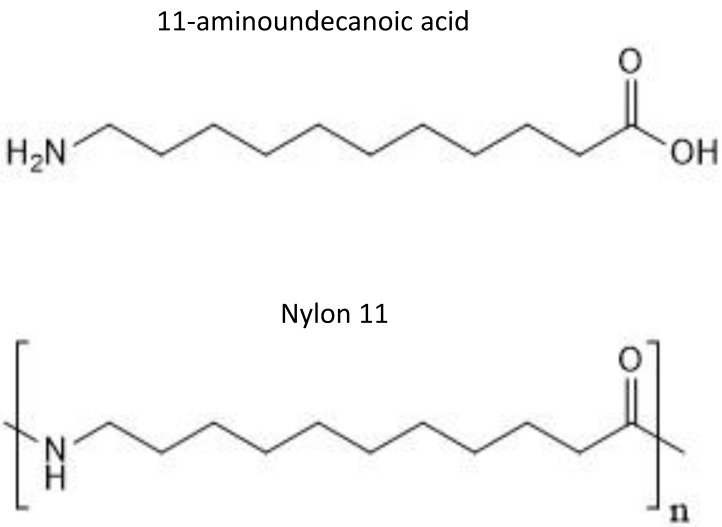
Chemical structure of 11-aminoundecanoic acid and nylon 11.

Nylon 11 is a common synthetic bioplastic that is used in the manufacture of a wide range of commercial products. Nylon 11 is synthesized by polymerization of 11-aminoundecanoic acid, which is derived from castor oil (Figure 1) 1. Polymers form extensive inter-polymer interactions, which result in the crystalline structure and strength desired for many commercial materials. These structures, on the other hand, make polymers largely resistant to biotic and abiotic degradation. Therefore, when accidentally released into the environment, they remain intact for a long period of time and pose a significant threat to various organisms.

As a safe and cost-effective means to remediate plastics in the environment, biodegradation has attracted attention in recent years. Biodegradation of polyesters (e.g. polyethylene, polyurethane, and poly(ethylene terephthalate) (PET)) and polyamides (e.g. nylons) by bacteria and fungi species have been investigated in the last couple of decades 2-5. Metabolic degradation of synthetic and biological polymers proceeds in two steps: extracellular fragmentations of polymers and then cellular metabolism of monomeric units.

Enzymes that hydrolyze synthetic polymers (plastics) have been identified 6, 7. Hydrolases, including esterases, lipases and cutinase hydrolyze ester linkage of PET 8-10. Extracellular poly(L-lactic acid) (PLA) depolymerase degrades PLA into lactic acid 7. Studies with nylon 6 polymerization by-products identified three enzymes that are involved in microbial degradation of a variety of nylon 6 oligomers, namely 6-aminohexanoate-cyclic-dimer hydrolase (EI), 6-aminohexanoate-dimer hydrolase (EII), and endotype 6-aminohexanoate-oligomer hydrolase (EIII) 11-14. In *Arthrobacter* sp. KI72, enzymes EI, EII, and EIII are encoded from genes, *nylA*, *nylB* and *nylC*, respectively 11.

In the plasmid of *Arthrobacter* sp. KI723T1, all three genes (*nylA, nylB* and *nylC)* encoding enzymes with nylon 6 hydrolase activity were discovered 15. These genes are also present in other nylon 6 degrading bacterial isolates belonging to a variety of taxa, including *Pseudomonas, Agromyces,* and* Kocuria* 16-18. In the genome of *Agromyces* sp. KY5R, *nylB* and *nylC* are present, but it lacks *nylA* 17. In *Pseudomonas* sp. NK87, in contrast, *nylA* and *nylB* are identified on the plasmids, but missing *nylC* 18.

The study of nylon 11 biodegradation is scarce. In a previous study by Kuo et al., nylon 11 was first treated with trifluoroacetic and blended with chitosan 19. The authors demonstrated that the mass of chitosan-nylon 11 mixture decreased after being buried in soil for 10 to 40 days, indicating the possibility that nylon 11 is also biodegradable. However, to the best of our knowledge, the isolation of organisms that degrade nylon 11 or direct evidence of biodegradation by microorganisms has not been reported.

In our current study, to gain insights into a nylon 11 biodegradation process, we isolated a bacterial strain that grows on 11-aminoundecanoic acid, a precursor of nylon 11 synthesis and putative depolymerization product of nylon 11, from nylon 11 enrichment culture. There has been no previous report of the isolation of 11-aminoundecanoic acid metabolizing bacterial strains. The draft genome and phylogenetic analysis of the strain revealed that it belongs to genus *Pseudomonas*. The content of its genome included genes encoding extracellular hydrolases and homologues of nylon 6 hydrolases. Based on these findings, we postulated that a nylon 11 in enrichment culture was first cleaved by extra cellular enzyme(s) to form 11-aminoundecanoic acid, and then organisms such as JB-G utilized the 11-aminoundecanoic acid as carbon and nitrogen sources for growth.

## Materials and Methods

### Media and growth conditions

Cells were grown on defined media devoid of carbon and nitrogen by modifying basal salts medium (BSM) 20. Specifically, ammonium chloride and nitrilotriacetic acid were omitted from the media (BSM-CN). Soil was collected from the Indiana University Kokomo campus. To construct nylon 11 enrichment culture, 1 g of soil and 1 g of nylon 11 pellets were added to 50 ml BSM-CN media. As a control, BSM-CN media containing succinate and nitrate as a carbon and nitrogen source was also created. After incubation at 30 °C with constant agitation at 150 rpm, cells were plated on BSM-CN agar medium supplemented with 11-aminoundecanoic acid as sole source of carbon and nitrogen.

For growth curve analysis, cells were transferred from overnight culture grown on BSM-CN media containing 11-aminoundecanoic acid (1%) to a fresh media, and grown at constant agitation at 150 rpm at 30 °C. The change in absorbance at 600 nm was monitored using the spectrophotometer. The growth was measured in triplicate.

### Genome determination

The genomic DNA was purified using E.Z.N.A. Stool DNA kit (Omega Bio-tek, Inc., GA). Genome sequences were obtained by Illumina Hiseq 2500 sequencer at Macrogen Inc. (MD, USA). The de novo genome assembly was performed by Velvet 1.2.07 21 and the graphical user interface Vague 22. The K-mer size was chosen by software based on the genome size of 6 M bases. Genome annotation was conducted by RAST 23.

### Phylogenetic analysis

Phylogenetic tree of 16S rRNA gene sequences was constructed via Maximum Likelihood method using MEGA6 with bootstrap values of 500 replications 24.

## Results and Discussion

**Figure 2:**
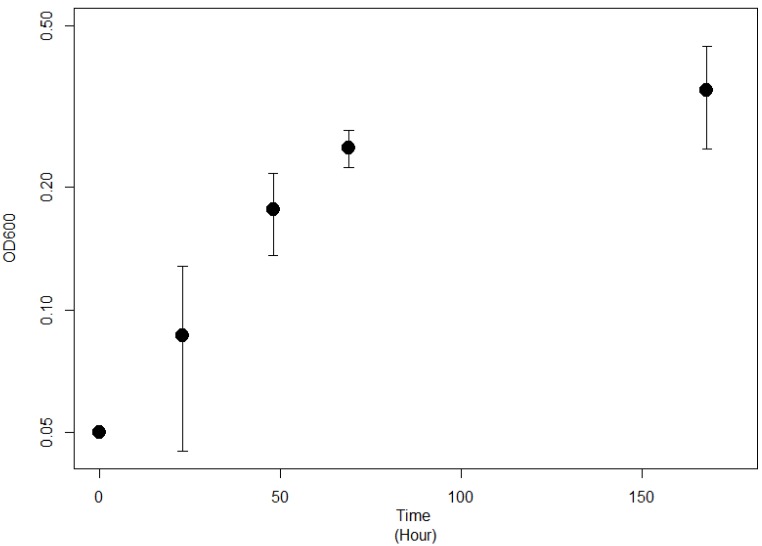
Growth curve of *Pseudomonas* sp. strain JG-B on 11-aminoundecanoic acid as sole source of carbon.

Strain JG-B was isolated from solid media containing 11-aminoundecanoic acid as a sole source of carbon and nitrogen. They were abundantly present on the solid media inoculated with nylon 11 enrichment culture but absent on the medium plated with cells from control enrichment culture. It also showed rapid growth on 11-aminoundecanoic acid in liquid culture (Figure 2). In order to rule out the possibility that the observed growth on 11-aminoundecanoic acid was due to the use of stored nitrogen in cells, growth in liquid culture was verified by repeated subculturing up to eight times. Atmospheric nitrogen was also a possible source of nitrogen for growth. Various growth tests were performed to eliminate this possibility. First, JG-B strain did not grow when carbon source alone (e.g. succinate) was added to the BSM-CN media (Table 1). Only when nitrogen was also provided as nitrate or as 11-aminoundecanoic acid, cells exhibited growth. Additionally, homologues of genes encoding known nitrogenase were not identified on the genome as described below.

Illumina sequencing of the genomic DNA produced a total 11.4 million reads which were *de novo* assembled into 90 contigs. The sequence has been deposited in NCBI GenBank under the accession number VOWX00000000. The total genome size is approximately 6 Mbp with 62.9 % GC content. It is composed of 5,367 coding sequences and 66 RNA genes (Table 2).

**Figure 3:**
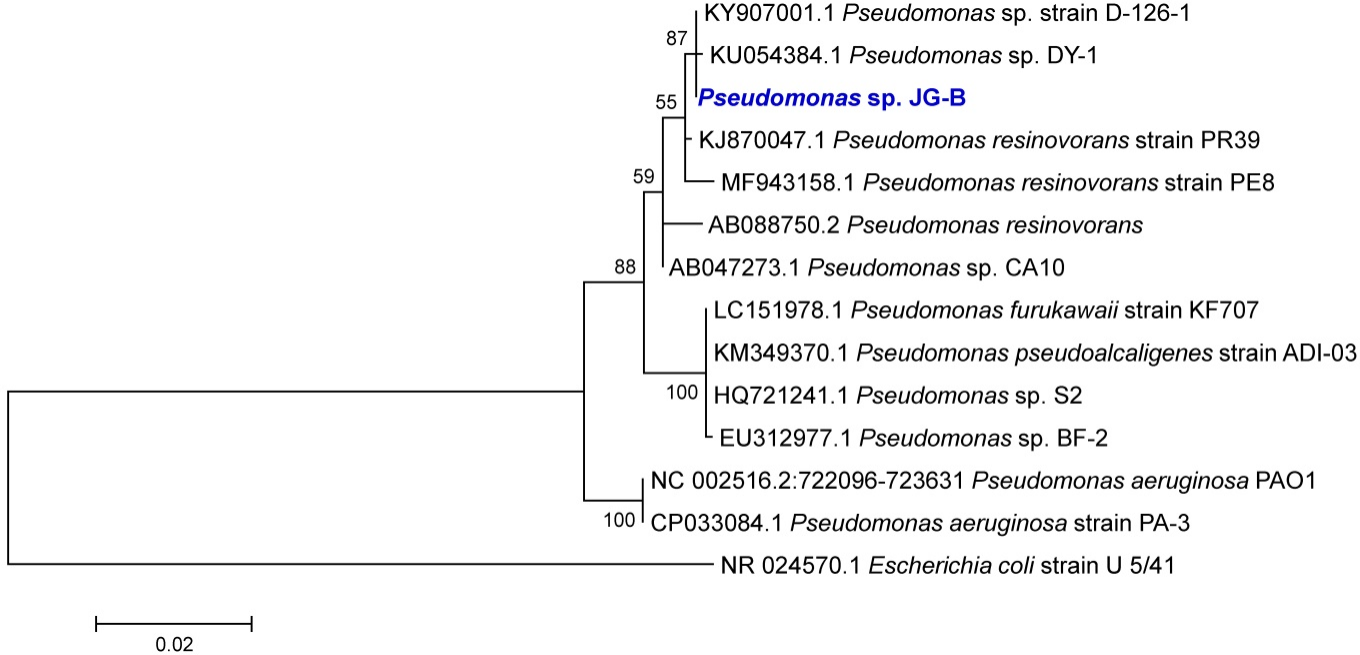
Phylogenetic tree of 16S rRNA gene sequences of *Pseudomonas* sp. strain JG-B and related strains. The tree was constructed using the maximum likelihood method. Numbers at nodes indicate bootstrap values. The scale bar represents nucleotide substitutions per site.

The phylogenetic analysis of the 16S rRNA gene sequences revealed that strain JG-B belongs to the genus *Pseudomonas*. Many *Pseudomonas* strains degrade a variety of aliphatic compounds 25, 26. A phylogenetic tree was constructed with sequences of *Pseudomonas* strains with known biodegradation abilities (Figure 3). Notably, 16S rRNA gene sequence of JG-B strain showed a high sequence identity (97.5%) to that of *P. aeruginosa* PAO1. While wildtype strain PAO1 does not metabolize nylon 6, Prijambada et al. successfully created a mutant that degraded nylon 6 oligomers via experimental evolution 27.

Metabolism of 11-aminoundecanoic acid has never been reported. A recent study of the cellular metabolism of 6-aminohexanoate (the hydrolysis product of nylon 6) identified involvement of 5-aminovalerate aminotransferase/ Gamma-aminbutyrate:alpha-ketoglutarate aminotransferase (NylD) 28. While 6-aminohexanoate and 11-aminoundecanoic acid differs significantly in the length of carbon chain (i.e. 6 vs 11), it is possible that it utilizes related enzymes and involves aminotransfer reaction. Blast analysis of NylD with JG-B showed that the JG-B genome contains a gene (FUT48_13600) which would encode a protein with 48 % protein sequence identity to NylD of *Arthrobacter* sp. KI72 (WP_079941468). Eleven additional genes encoding aminotransferase with a high similarity to *Arthrobacter* sp. KI72 NylD (i.e. >27% protein sequence identity) were also present on the strain JG-B's genome. Further research will be needed to decipher if any of these enzymes are involved in the 11-aminoundecanoic acid metabolism.

**Figure 4:**
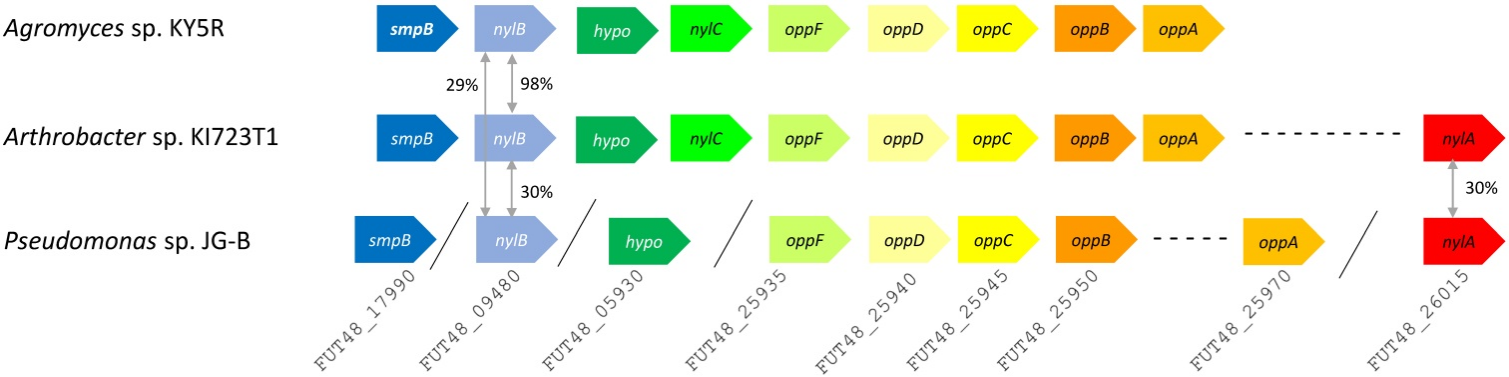
Putative nylon 11 degradation genes in *Pseudomonas* sp. strain JG-B and nylon 6 degrading bacteria. Abbreviations for genes and encoded proteins: *smpB*: tmRNA-binding protein; *nylB*: 6-aminohexanoate dimer hydrolase (EII); *hypo*: hypothetical protein; *nylC*: 6-aminohexanoate-oligomer hydrolase (EIII); *oppF*: oligopeptide transport ATP-binding protein; *oppD*: oligopeptide transport ATP-binding protein; *oppC*: oligopeptide transport system permease protein; *oppB*: oligopeptide transport system permease protein; *oppA*: periplasmic oligopeptide binding protein; and *nylA*: 6-aminohexanoate-cyclic-dimer hydrolase (EI). A symbol / indicates when genes are located on different contigs. The values represent protein sequence identity between the pair of orthologs.

A variety of genes encoding hydrolases are also found on JG-B's genome, including a homologue of an extracellular lipase gene from *Pseudomonas wisconsinensis* (FUT48_16475), predicted extracellular lipase/esterase (FUT48_02000), and nylon 6 hydrolases (FUT48_26015 and FUT48_09480 encoding NylA and NylB, respectively) (Figure 4). NylA and NylB both shared 30 % protein sequence identity with corresponding proteins of *Arthrobacter* sp. KI723T1. A homologue of gene encoding NylC was absent on JG-B strain. In both *Arthrobacter* and *Agromyces* sequences, genes encoding subunits for oligopeptide permease (Opp) and tmRNA binding small protein B (SmpB) are present in the same region as genes encoding NylB and NylC. Both types of genes were also found on the genome of JG-B but they do not appear to be located in the neighboring region.

NylA is amidase, related to Asp-tRNAAsn/Glu-tRNAGln amidotransferase A subunit 29. Through structural studies and mutagenesis of NylA in *Arthrobacter*, three residues at catalytic center (Ser^174^, Ser^150^ and Lys^72^) and a residue (Cys^316^), that are proposed to be involved in specific binding of NylA to its substrate 6-aminohexanoate cyclic dimer, were identified. Despite low sequence identity with *Arthrobacter* NylA (30%), JG-B's NylA contains identical catalytic residues, suggesting a conserved catalytic mechanism. In contrast, Cys^316^ is replaced by alanine in JG-B's NylA, suggesting different substrate specificity.

NylB is a penicillin-recognizing serine hydrolase sharing high structural similarity to DD-peptidase and carboxylesterase (EstB) 30. In this penicillin recognizing family of serine hydroxylases, a serine residue of Ser-X-X-Lys motif functions as nucleophile in catalysis 30. The motif was identified in the JG-B's NylB protein sequence. Like related NylB proteins, JG-B's NylB lacks other motifs present in EstB (e.g. Gly-X-Ser-X-Gly motif) and class A β-lactamases (e.g. KTG box). These observations suggest that catalytic residues appear to be located within the Ser-X-X-Lys motif.

Additionally, NylB from JG-B and *Arthrobacter* strain K172's EII enzyme share another active site residue (Y^215^ in 1WYB). However, residues that were shown to be important for 6-aminohexanoate linear dimer hydrolyzing activity were different or missing in JG-B's NylB sequence. Asp^181^ has no corresponding residue in JG-B's NylB, and Asn^266^ is replaced by Ser in JG-B 30. These differences may reflect the divergences in their substrate(s).

In summary, we have isolated a *Pseudomonas* sp. strain JG-B that can grow on 11-aminoundecanoic acid as a sole source of carbon and nitrogen. Through genomic analysis, we identified genes that may be involved in the metabolism of 11-aminoundecanoic acid as well as its polymer nylon 11. We are currently investigating the nylon 11 degradation ability of this organism.

## Figures and Tables

**Table 1:** Growth of *Pseudomonas* sp. strain JG-B with various carbon and nitrogen sources.

Substrates	Carbon source	Nitrogen source	Growth
11-aminoundecanoic acid	11-aminoundecanoic acid	11-aminoundecanoic acid	Growth
Succinate	Succinate	No	No growth
Succinate/nitrate	Succinate	Nitrate	Growth

**Table 2:** Genomic features of *Pseudomonas* sp. strain JG-B

Features	
Total length (bp)	5,991,756
N50	279,576
GC content (%)	62.9
Total number of genes	5,588
Protein coding gene (CDS)	5,367
rRNA genes	3
tRNA genes	59
ncRNA genes	4
